# Supplementary material for: A single-center retrospective study of ectopic lymphoid tissues in idiopathic membranous nephropathy: clinical pathological characteristics and prognostic value
Source: PeerJ. 2024 Dec 9;12:e18703. doi: 10.7717/peerj.18703 (PMC11636532; doi:10.7717/peerj.18703)
Supplement: Supplemental Information 2 [file peerj-12-18703-s002.docx]

**Supplemental Table 1 Induction treatment response and renal outcomes of patients with nephrotic syndrome.**

|  | **Low-grade group (n=23)** | **Intermediate-grade group**  **（n=31）** | **High-grade group（n=18）** | ***P*-value** |
| --- | --- | --- | --- | --- |
| Immunosuppressive therapy during the first 6 months, n (%) | 21 (91.3) | 26 (83.9) | 17 (94.4) | 0.635 |
| Treatment response |  |  |  | 0.154 |
| Non-remission, n (%) | 7 (30.4) | 12 (38.7) | 12 (66.7) | 0.054 |
| Partial remission, n (%) | 9 (39.1) | 14 (45.2) | 4 (22.2) | 0.287 |
| Complete remission, n (%) | 7 (30.4) | 5(16.1) | 2 (11.1) | 0.348 |
| Renal outcomes |  |  |  |  |
| ESRD, n (%) | 0 (0) | 0 (0) | 3 (16.7)^a,b^ | 0.014^＊^ |
| Doubling of Scr, n (%) | 1 (4.3) | 6 (19.4) | 2 (11.1) | 0.287 |

Values for categorical variables are given as count (percentage). ESRD, end-stage renal disease; Scr, serum creatinine.

^＊^Two-tailed *P* < 0.05.

^a^ *P* < 0.05 *vs* low-grade group, ^b^ *P* < 0.05 *vs* intermediate-grade group.
